# Women suffering from chronic rhinosinusitis in Norway are more likely to take sick leave

**DOI:** 10.1371/journal.pone.0313122

**Published:** 2024-11-01

**Authors:** Ulrika K. E. Clarhed, Linus Schiöler, Kjell Torén, Anne Kristin M. Fell, Johan Hellgren

**Affiliations:** 1 Department of Otorhinolaryngology, Institute of Clinical Sciences, The Sahlgrenska Academy, University of Gothenburg, Gothenburg, Sweden; 2 Department of Otorhinolaryngology, Capio Lundby Hospital, Gothenburg, Sweden; 3 Occupational and Environmental Medicine, Institute of Medicine, The Sahlgrenska Academy, University of Gothenburg, Gothenburg, Sweden; 4 Department of Occupational and Environmental Medicine, Telemark Hospital, Skien, Norway; 5 Department of Community Medicine and Global Health, Institute of Health and Society, University of Oslo, Oslo, Norway; 6 Department of Otorhinolaryngology, Region Västra Götaland, Sahlgrenska University Hospital, Gothenburg, Sweden; University Putra Malaysia, MALAYSIA

## Abstract

**Background:**

Chronic rhinosinusitis (CRS) decreases the quality of life and affects the working life of sufferers. There is a scarcity of studies of how CRS affects sick leave at the population level, particularly for women.

**Materials and methods:**

Data from questionnaires were collected in Telemark, Norway in 2013 (N = 15,484) and again in 2018 (N = 13,966). Odds ratios with 95% confidence intervals (CI) for having sick leave in the last 12 months, adjusted for sex, asthma, smoking and age, were calculated, as well as the relationship to occupational groups. Comparisons were made between women and men.

**Results:**

Subjects with CRS had 64% increased odds for taking sick leave compared to subjects without CRS (OR 1.64, 95% CI 1.45–1.85) in 2013, with similar results in 2018 (OR 1.60, 95% CI 1.41–1.81). Women with CRS were almost twice as likely to take sick leave than men with CRS (OR 1.96, 95% CI 1.56–2.46) in 2013. Sick leave was more common in subjects with CRS in some occupational groups.

**Conclusion:**

CRS is a chronic and debilitating disease that appears to affect sick leave on a population level, with women being more affected than men. Optimised treatment for CRS might reduce sick leave and associated costs.

## Introduction

Chronic rhinosinusitis (CRS) is an inflammatory disorder of the nose and paranasal sinuses that is characterised by nasal congestion and/or nasal discharge, facial pain and impaired sense of smell [1]. CRS has reported prevalence rates of around 11% in Europe [2], around 12% in the United States [3], and 2%–28% in Asia [4]. Patients with CRS experience a negative impact on their health-related quality of life [5, 6]. CRS affects social functioning to the same extent as does angina or chronic heart failure [5]. Some patients with CRS experience a decrease in health-related quality of life similar to that experienced by patients with head and neck cancer or hysterectomy [7]. One of the most important factors that affects the quality of life of patients with CRS is poor sleep quality [7]. CRS mainly affects people of working age [3], and as such the quality of sleep is especially important, as lack of sleep also affects negatively the ability to work. A Swedish study from 2011 that comprised 207 patients with recurrent acute rhinosinusitis and CRS who were referred for functional endoscopic sinus surgery illustrated the significant impact of CRS on the health-related quality of life of the subjects [8]. Those patients were also asked to report on how many days they had been absent from work in the last 12 months due to sinus problems. In this selected group of CRS patients who were undergoing surgery, 57% reported absenteeism (missed workdays) due to sinus problems. Thus, it is clear that the economic burden of CRS is significant. It has been estimated that the CRS-related economic burden in the US was approximately 30 billion US dollars per year in 2011 [9]. In Europe, with a larger population, the corresponding amount is presumably even higher.

A few previous studies have looked at how CRS is associated with limitations regarding the ability to work [8, 10]. Pandrangi and colleagues studied patients with refractory CRS who were undergoing surgery and found significant median post-operative work productivity and activity gains of 10% and 20%, respectively [10]. These studies have focused on patients with refractory CRS who were elected for surgery. However, there is little information on how CRS affects the ability to work in a general population, despite it being a commonly diagnosed illness even outside of Ear-Nose-Throat clinics [2, 11]. In one study [12], subjects with CRS in the general population had significantly increased odds for limitations related to work and activities, as well as social limitations, as compared with subjects without CRS. However, that study lacks information regarding sick leave.

We have previously studied a large, prospective population cohort from the south-eastern part of Norway regarding risk factors for the development of CRS in the working environment, and have shown that many exposures increase the risk of developing CRS [11]. It is still not known, however, how CRS affects the ability to work in the general population, as many patients are never subjected to sino-nasal surgery or an otorhinolaryngologic assessment. The present study is part of the Telemark population study and is, to the best of our knowledge, the first population-based study assessing how CRS affects sick leave in the general population, and particularly for women.

## Materials and methods

### Study population and design

The Telemark Study in Telemark County, Norway began in 2013 when 50,000 randomly selected individuals in the age range of 16–50 years were invited to participate in the questionnaire-based study. The questions concerned CRS, asthma, smoking, present and past occupations, and sick leave, amongst others. The same questionnaire was sent out again in 2018. For a more comprehensive description of the study, the reader is directed to our previous publications [11, 13].

**Fig 1:**
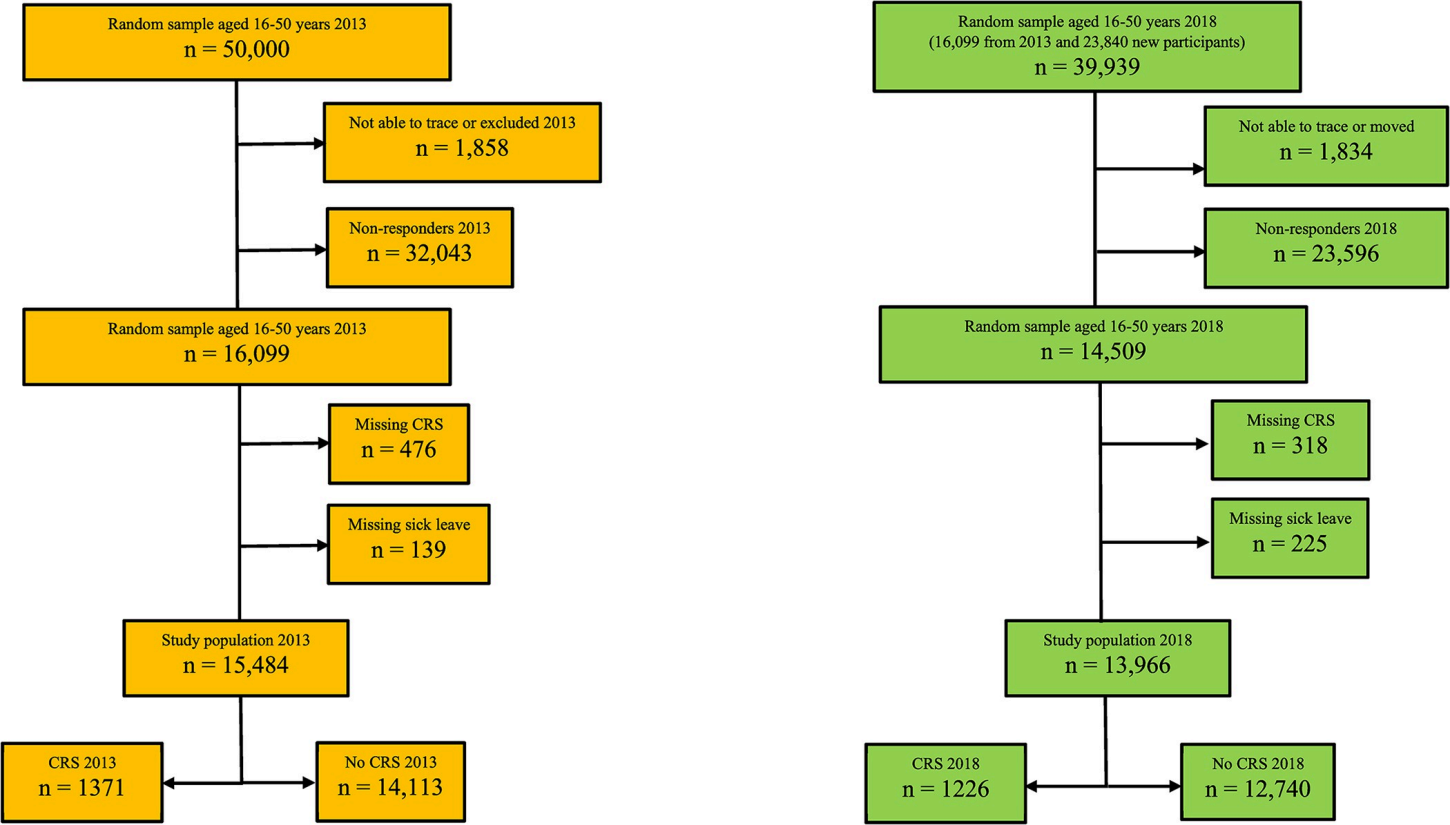
Flow-chart of the study populations in 2013 (yellow) and 2018 (green).

In total, 16,099 subjects responded to the questionnaire in 2013. The questionnaire was sent out again in 2018 to the same 16,099 subjects, as well as to 23,840 new subjects who fulfilled the inclusion criteria. In total, 14,509 subjects answered the questionnaire in 2018. The present study was conducted as two cross-sectional studies in 2013 and in 2018, respectively. Subjects were divided into those fulfilling the requirements for the diagnosis of CRS and those who did not (no CRS). A flow-chart of the study population is provided in Fig 1.

CRS was defined according to the European position paper on rhinosinusitis and nasal polyps (EPOS) criteria for epidemiological questionnaire-based studies as: inflammation of the nose and the paranasal sinuses characterised by the presence of two or more symptoms for ≥12 weeks, with one of these symptoms being nasal blockage/obstruction/congestion or nasal discharge (anterior/posterior nasal drip), called “major symptoms”, as well as additional symptoms, such as facial pain/pressure and/or a reduction in or loss of the sense of smell, called “minor symptoms” [1].

Sick leave in the last year was defined as a positive answer to the question *“Have you been on sick leave over the course of the last 12 months*?*”*. Subjects were considered to have asthma if they responded yes to the question *“Has a doctor ever diagnosed you with asthma*?*”*. Subjects were registered as not having asthma if they answered no to either the question *“Has a doctor ever diagnosed you with asthma*?*”* or *“Do you have*, *or have you ever had*, *asthma*?*”*. Age was analysed as a continuous variable. Smoking was assessed using the following questions; 1)*“Do you smoke daily (even if you only smoke a few cigarettes*, *cigars or a pipe daily)*?*”* 2)*“Do you smoke only occasionally (not daily*, *but weekends*, *party smoking and the like*?*”* 3)*“Did you use to smoke previously*?*”*. A positive answer to questions 1 and/or 2 were registered as current smoking and a positive answer to question 3 was registered as past smoking.

In a sub-analysis, we attempted to grade the severity of CRS in relation to the number of CRS-related symptoms in each individual [14]. Subjects were divided into the following four groups: 1) subjects who reported no sinus symptoms at all (*no CRS-related symptoms*); 2) subjects who reported one or more sinus symptoms but did not meet the requirements for a diagnosis of CRS (*CRS-related symptoms*); 3) subjects who fulfilled the CRS criteria (*CRS*); and 4) subjects who fulfilled the CRS criteria and reported additional sinus symptoms (*CRS+*).

Subjects reported their occupational history and current occupation in 2013 and 2018. These were then classified by trained research assistants using the International Standard Classification of Occupations (ISCO-88) coding system into ten different groups [15, 16]. In order to achieve larger groups with more statistical power, these ten groups were merged into five larger groups: 1) *Legislators*, *senior officials and managers* and *Professionals* and *Armed forces*; 2) *Technicians and associate professionals*; 3) *Clerks* and *Service workers and shop and market sales workers*; 4) *Skilled agriculture and fishery workers* and *Craft and related trade workers*; and 5) *Plant and machine operators and assemblers* and *Elementary occupations*. Groups 1–3 contain occupations that are defined as *white collar* and Groups 4–5 contain occupations that are defined as *blue collar* [17].

Written and spoken information was given to all subjects and informed consent was collected. The study was approved by the Regional Committee for Medical and Health Research Ethics in Norway (2012/1665/REK sør-øst D).

### Statistical analyses

The subjects were divided into those who fulfilled the criteria for CRS and those who did not (no CRS). The associations of CRS and CRS symptoms with sick leave were investigated using multivariable logistic regression models, from which p-values, odds ratios (OR) and 95% confidence intervals (CIs) were calculated. Age, sex, asthma and smoking status were included in all the models as potential confounders, unless noted otherwise. Age was included as a cubic restricted spline with knots placed at the 10^th^, 50^th^ and 90^th^ percentile. Interactions were included to calculate specific estimates for sub-groups, e.g., to estimate the OR for women with CRS vs men with CRS, the model included CRS, sex and CRS×sex. The Cochran-Armitage trend test was used to investigate trends in the association between number of sinus symptoms and sick leave. The confidence and significance levels were set to 0.95 and 0.05, respectively.

## Results

A description of the study population with regards to age, smoking habits, sex and asthma can be found in Table 1. The prevalence of CRS was 9% in both 2013 and 2018. The distributions of age and sex in the CRS and non-CRS group were similar in both 2013 and 2018.

**Table 1:** Description of the study populations at baseline in 2013 (N = 15,484) and at follow-up in 2018 (n = 13,966) with regard to the prevalence of CRS, age, smoking habits, sex and asthma.

		2013			2018		
		CRS	No CRS	Total	CRS	No CRS	Total
**N** **(%)**		1,371 (9)	14,113 (91)	15,484	1,226 (9)	12,740 (91)	13,966
**Age, years**							
**Median (interquartile range)**		39 (29–45)	38 (26–45)	38 (27–45)	45 (34–51)	45 (35–51)	45 (35–51)
**Smoking** **N (%)**							
	**Never smoker**	616 (45)	8,009 (57)	8,625 (56)	607 (50)	7,290 (57)	7,897 (57)
	**Past smoker**	310 (23)	2,867 (20)	3,177 (21)	346 (28)	3,267 (26)	3,613 (26)
	**Current smoker**	440 (32)	3,180 (23)	3,620 (24)	273 (22)	2,150 (17)	2,423 (17)
**Sex** **N (%)**							
	**Female**	752 (55)	7,852 (56)	8,604 (56)	702 (57)	7,344 (58)	8,046 (58)
	**Male**	619 (45)	6,261 (44)	6,880 (44)	524 (43)	5,396 (42)	5,920 (42)
**Asthma** **N (%)**							
	**Yes**	380 (29)	1,425 (10)	1,815 (12)	336 (28)	1,374 (11)	1,710 (12)
	**No**	947 (71)	12,453 (90)	13,400 (88)	884 (72)	11,296 (89)	12,180 (88)

**Fig 2:**
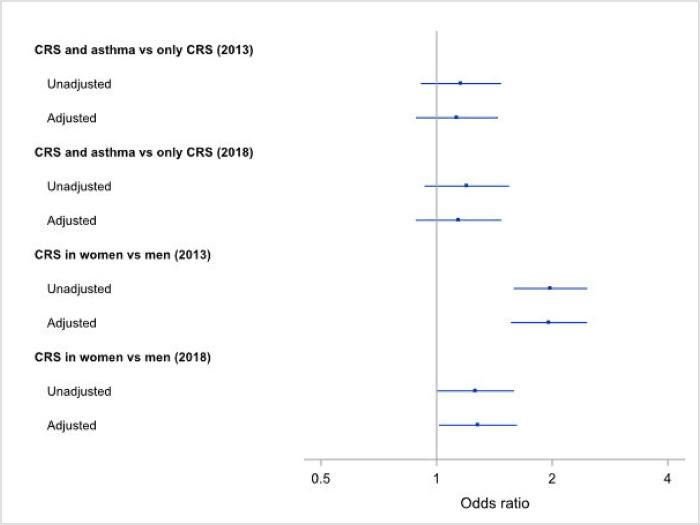
Multivariable regression analysis of sick leave in the last 12 months for the 2013 and 2018 studies, adjusted for smoking, age and sex (CRS and asthma vs only CRS) and adjusted for smoking, age and asthma (CRS in women vs CRS in men). Shown are the odds ratios (ORs).

The odds for taking sick leave in the last 12 months were significantly increased in subjects with CRS in both 2013 (OR 1.64, 95% CI 1.45–1.85) and 2018 (OR 1.60, 95% CI 1.41–1.81) (Table 2). When subjects with CRS and asthma were compared to those having only CRS there was no significant difference in the odds of taking sick leave (Table 3 and Fig 2). Women with CRS had an increased odds for sick leave compared to men with CRS in 2013, both without adjustment (OR_unadj_ 1.98, 95% CI 1.59–2.47) and with adjustment (OR_adj_ 1.96, 95% CI 1.56–2.46) for age, asthma and smoking habits. Women with CRS also had an increased odds for sick leave compared to men with CRS in 2018, both without adjustment (OR_unadj_ 1.26, 95% CI 1.00–1.59) and with adjustment (OR_adj_ 1.28, 95% CI 1.01–1.62) for age, asthma and smoking habits (Table 3 and Fig 2).

**Table 2:** Multivariable regression analysis of sick leave in the last 12 months in the 2013 and 2018 studies for subjects with CRS compared to subjects without CRS, adjusted for age, sex, asthma and smoking. Shown are the odds ratios (OR) with 95% confidence intervals (95% CI).

	OR	95% CI	P-value
**Sick leave 2013**	1.64	1.45–1.85	< .0001
**Sick leave 2018**	1.60	1.41–1.81	< .0001

**Table 3:** Analysis of sick leave in the last 12 months in the 2013 and 2018 studies for subjects with CRS and asthma and only CRS, respectively, and for CRS in women and CRS in men, respectively (OR_unadj_). Multivariable regression analysis of sick leave in the last 12 months in the 2013 and 2018 studies, with adjustment for smoking, age and sex (CRS and asthma vs only CRS) and adjustment for smoking, age and asthma (CRS in women vs CRS in men) (OR_adj_). Shown are the odds ratios (OR) with 95% confidence intervals (95% CI).

	OR		95% CI	P-value
**CRS and asthma vs only CRS (2013)**				
	OR_unadj_	1.16	0.91–1.47	0.24
	OR_adj_	1.13	0.88–1.45	0.34
**CRS and asthma vs only CRS (2018)**				
	OR_unadj_	1.20	0.93–1.55	0.16
	OR_adj_	1.14	0.88–1.47	0.32
**CRS in women vs men (2013)**				
	OR_unadj_	1.98	1.59–2.47	< .0001
	OR_adj_	1.96	1.56–2.46	< .0001
**CRS in women vs men (2018)**				
	OR_unadj_	1.26	1.00–1.59	0.05
	OR_adj_	1.28	1.01–1.62	0.04

**Fig 3:**
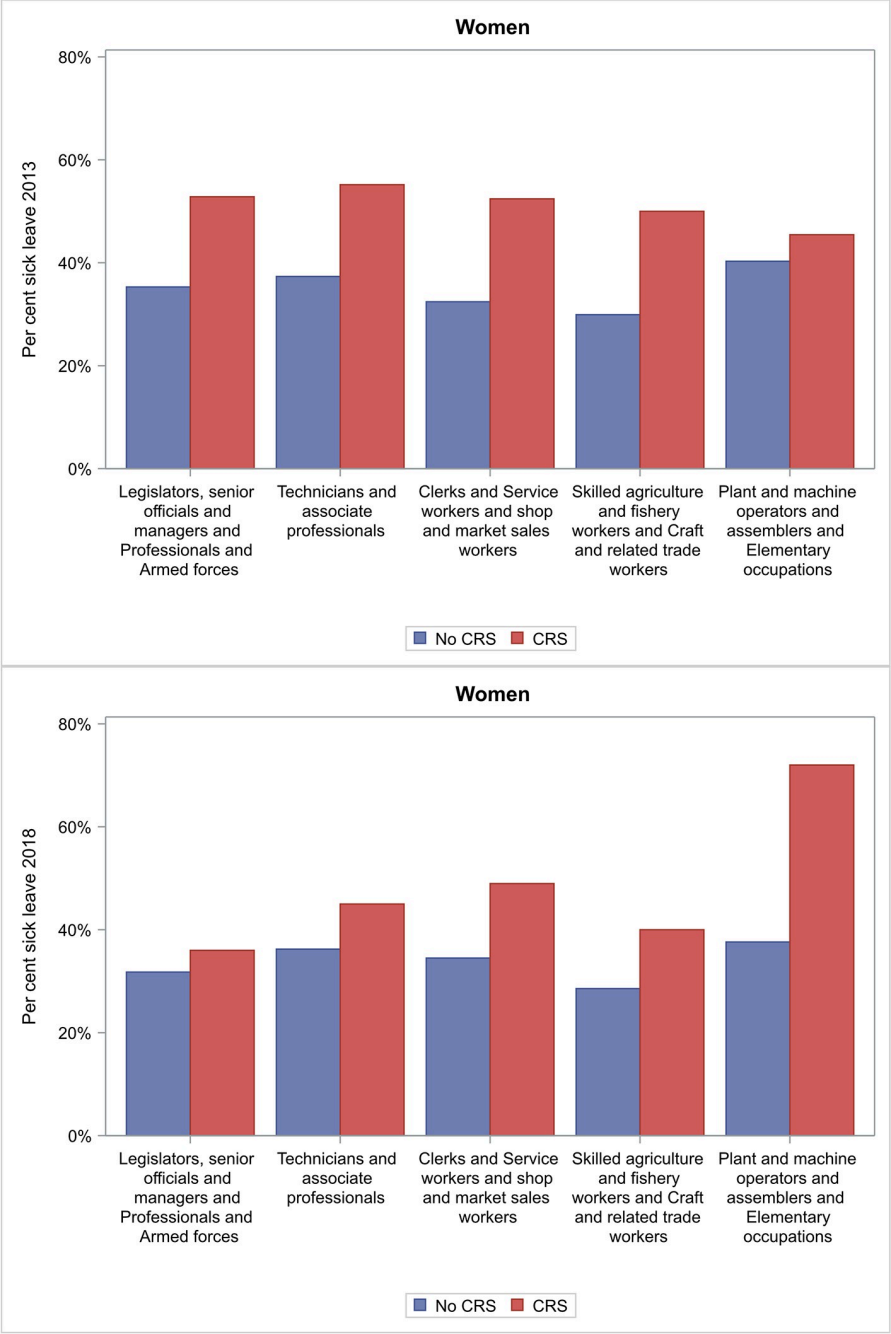
Occupational groups for women with CRS and women with no CRS in 2013 and 2018 who reported sick leave in the last 12 months.

In 2013 women with CRS reported having sick leave during the last year significantly more frequently compared to women without CRS in the following occupational groups: *Clerks* and *Service workers and shop and market sales workers*; *Legislators*, *senior officials and managers* and *Professionals* and *Armed forces*; and *Technicians and associate professionals* (Fig 3).

In 2018 women with CRS reported having sick leave during the last year significantly more frequently compared to women without CRS in the following occupational groups: *Clerks* and *Service workers and shop and market sales workers*; *Plant and machine operators and assemblers* and *Elementary occupations*; and *Technicians and associate professionals* (Fig 3).

**Fig 4:**
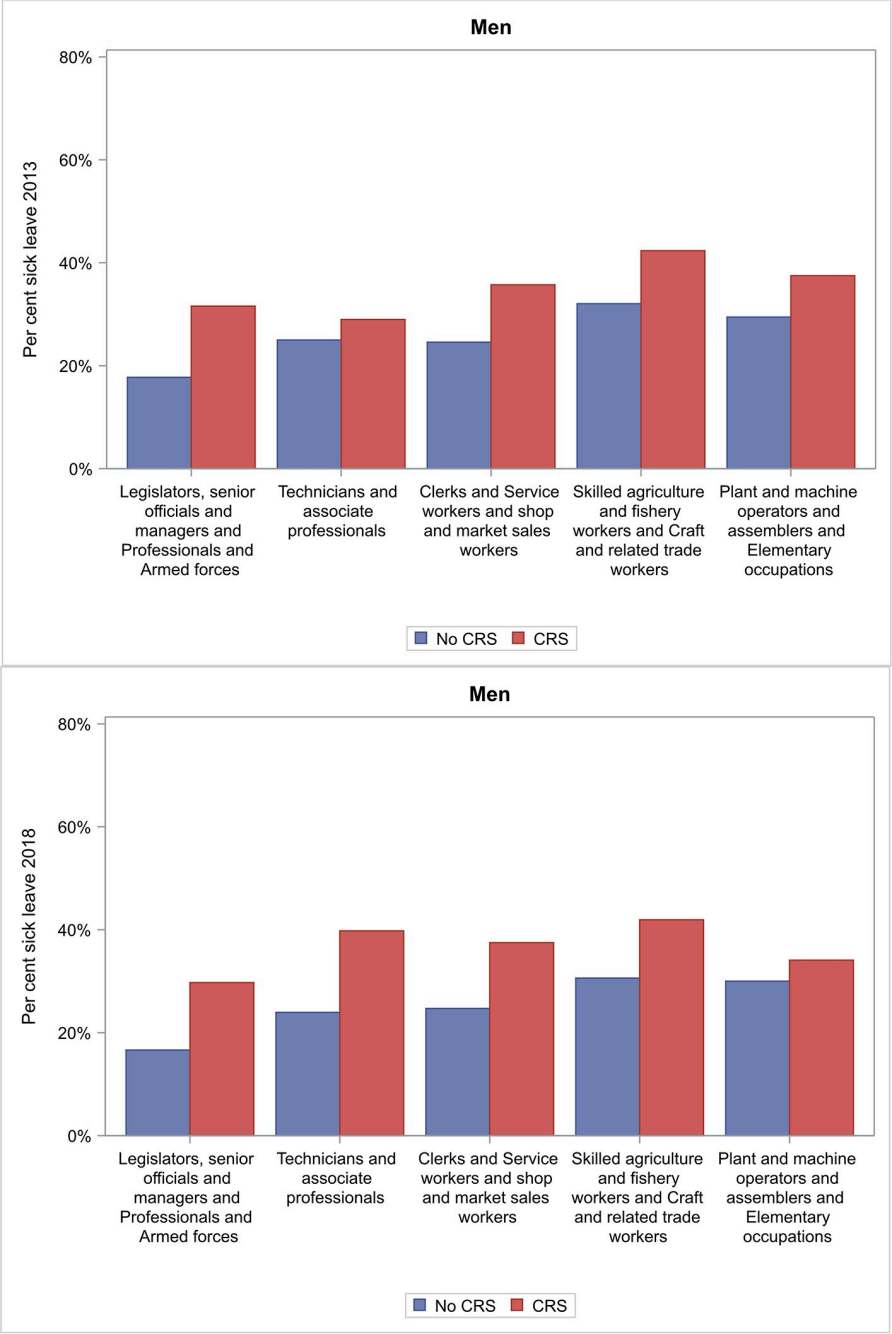
Occupational groups for men with CRS and men with no CRS in 2013 and 2018 who reported sick leave in the last 12 months.

In 2013 men with CRS reported having sick leave during the last year significantly more frequently compared to men without CRS in the following occupational groups: *Legislators*, *senior officials and managers* and *Professionals* and *Armed forces; Skilled agriculture and fishery workers* and *Craft and related trade workers* (Fig 4).

In 2018 men with CRS reported having sick leave during the last year significantly more frequently compared to men without CRS in the following occupational groups: *Legislators*, *senior officials and managers* and *Professionals* and *Armed forces; Skilled agriculture and fishery workers* and *Craft and related workers*; and *Technicians and associate professionals* (Fig 4).

**Fig 5:**
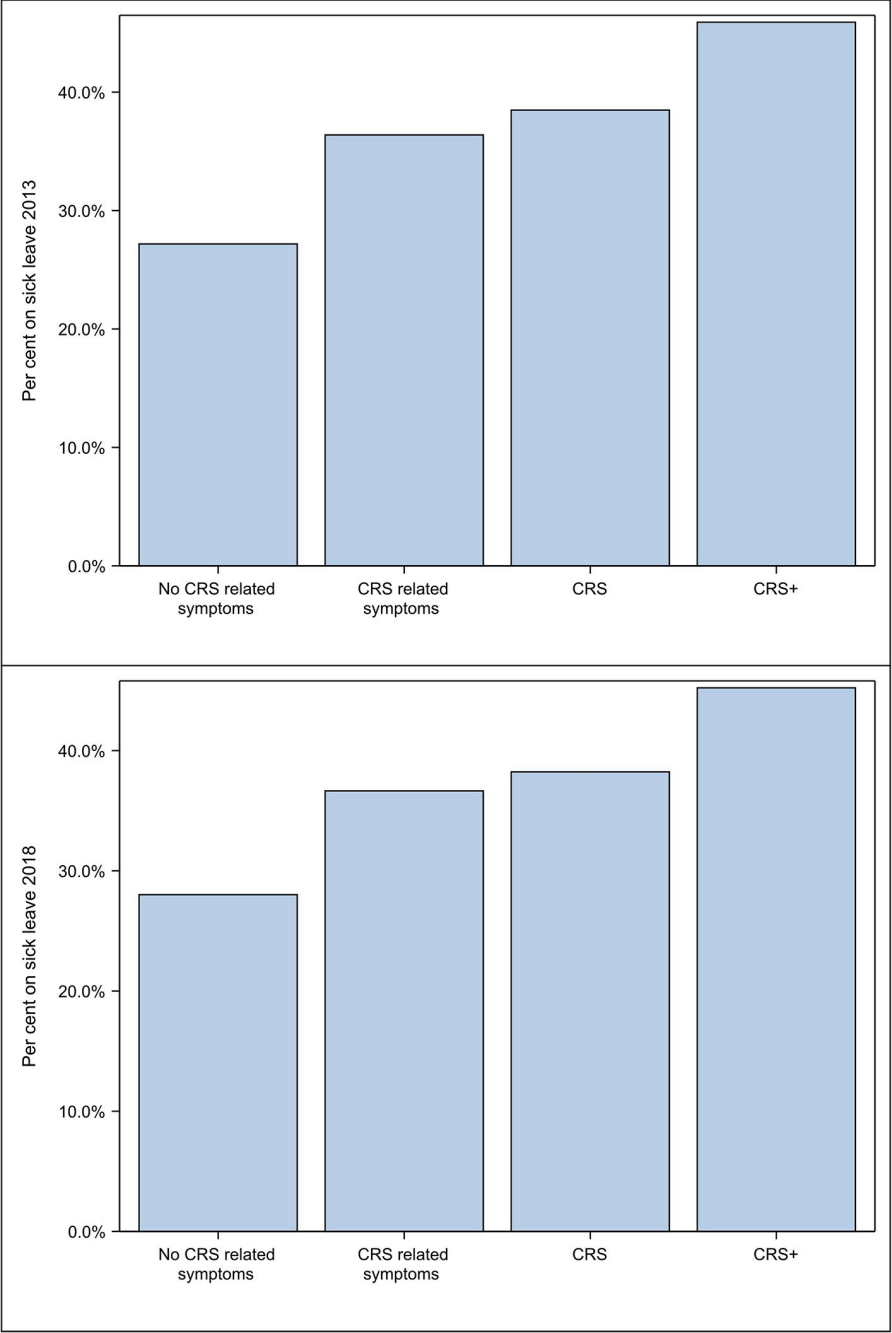
Compiled sinus symptoms and per cent sick leave in 2013 and 2018.

Sinus symptoms were also compiled and assessed in relation to sick leave in the last 12 months in both the 2013 and 2018 studies. The percentage of persons taking sick leave increased significantly with increasing number of sinus symptoms (p<0.0001) (Fig 5).

## Discussion

We found that subjects with CRS consistently had higher odds of taking sick leave during the past year compared to subjects without CRS, both at a median age of 39 years in 2013 (OR 1.64, 95% CI 1.45–1.85) and at a median age of 45 years in 2018 (OR 1.60, 95% CI 1.41–1.81) (Table 2 and Fig 2). Women had an almost two-fold higher odds of sick leave compared to men in 2013 (Fig 2), and sick leave was more common for subjects with CRS than for subjects without CRS in some occupational groups (Figs 3 and 4). This illustrates the seriousness of the disease and how its chronic nature can affect working life on a population level, although we have not studied if there is a causal linkage.

CRS is a costly disease with significant economic impacts on both society and the individual patient [18, 19]. A few studies have focused on limitations related to the ability to work for patients with refractory CRS in a surgical setting, although little is known about the same parameter for subjects with CRS in the general population. Bhattacharyya has studied how CRS affects the work and recreational time of subjects at a population level [12] and has reported that subjects with CRS have significantly increased odds of experiencing limitations related to work (OR 1.50, 95% CI 1.13–1.98) and activities (OR 1.54, 95% CI 1.20–1.99), as well as social limitations (OR 1.49, 95% CI 1.14–1.97). These results do not specify the ways in which these limitations are manifested, and there is a lack of data regarding sick leave. Our findings in the present study imply that sick leave is one such limitation regarding work for subjects with CRS. This affects the individual with CRS, who may be at risk of missing career opportunities, having a lower wage, and possibly losing their job.

To the best of our knowledge, we are the first to study the types of work that subjects with CRS who have had recent sick leave report on a population level. Women with CRS reported having sick leave during the last year significantly more frequently compared to women without CRS in several occupational groups (Fig 3). These occupational groups include cooks and hairdressers, amongst others. In this context, we have previously reported an association between new-onset CRS and self-reported occupational exposure to hair-care products and cooking fumes [20]. As those previous results may be affected by information bias, the use of occupational titles is also important. Nonetheless, specific exposures appear to be related to disease progression. Other factors, such as non-occupational allergy and smoking, are also implicated in the development of CRS [2, 21]. The results of the present study indicate that some occupational exposures, for example hair-care products and cooking fumes, cause a deterioration in CRS, with reduced disease control and, consequently, more frequent sick leave.

Both women and men with CRS reported having sick leave in the last year significantly more frequently compared to subjects without CRS in both white-collar and blue-collar occupations. There were no obvious trends indicating more frequency of sick leave in one group than in another. The fact that sick leave was increased broadly across many different occupational groups may reflect that CRS affects subjects at all levels of society, regardless of occupation. It may be that only certain occupations contribute to the development of CRS, whereas the disease affects all patients regardless of occupation. Previous studies have highlighted the importance of sleep when it comes to quality of life for patients with CRS [7]. Poor-quality sleep, linked in part to CRS, could have negative effects on both white-collar and blue-collar occupations. CRS encompasses benign albeit cumbersome symptoms, such as nasal congestion or discharge, loss of smell, and facial pain, and it may be that once a subject has the disease there is a drop in their productivity at work, regardless of occupation or what caused the disease.

We also conducted a sub-analysis of compiled sinus symptoms and sick leave (Fig 5). This illustrates how sick leave increased with increasing number of sinus symptoms, indicating a possible correlation and that the disease burden increases with increasing number of CRS symptoms.

Another interesting finding is that women have an almost two-fold higher odds of sick leave when afflicted with CRS, as compared to men with CRS (OR 1.96, 95% CI 1.56–2.46, Fig 2). Chronic rhinosinusitis with nasal polyps is more common in men [22, 23], although this does not appear to have had any noticeable effect on sick leave in our study. There are differences in the normal nasal anatomy and physiology between women and men, in that women have a significantly smaller nasal surface area and volume [24]. One in five women may also be affected by pregnancy rhinitis, the pathogenesis of which remains unclear [25]. One hypothesis is that due to the normal nasal anatomical and physiological differences between women and men, CRS in women is perceived as being more severe than in men and, consequently, results in more sick leave. There is also a pronounced difference in the results from 2013 with a median age of 39 years (OR 1.96, 95% CI 1.56–2.46) and those from 2018 with median age of 45 years (OR 1.28, 95% CI 1.01–1.62). We can only speculate regarding the reason for this. Women aged 39 years may have younger children and, consequently, have a greater need for sick leave due to common and frequent childhood infections. This in addition to a CRS diagnosis may explain the increased frequency of sick leave for women.

When analysing the effect of asthma on top of CRS regarding sick leave, there appears to be no surplus effect (Fig 2 and Table 3). Subjects with asthma in the Telemark population generally have mild asthma, and this could be one reason for the lack of effect of asthma. However, this finding is interesting and may indicate that the symptoms caused by asthma, such as reduced physical performance, may be easier to cope with for the individual subject as opposed to the constant congested nose and lack of sleep caused by CRS [1, 7]. Further studies of this aspect are warranted.

The Telemark population study encompasses a large population of subjects of working age, and this is a strength because it enables us to study how CRS affects sick leave on a population level. There are, however, some limitations that should be addressed. The study is questionnaire-based, so there is no clinical nasal examination. The symptom criteria used for self-reported CRS in this study are, however, in line with current guidelines for epidemiological studies [1]. While there may as a result be some over-estimation of the prevalence of CRS, this has been addressed in previous studies [26, 27], with findings of reasonable correlation. The response rate in the Telemark study was 33% in 2013 and 38% in 2018. A non-responder study was conducted in 2016 and the prevalence for physician-diagnosed asthma was similar amongst responders and non-responders [13]. Another limitation of the study is that the specific reason for sick leave is not known. There are co-morbidities to be considered. However, there are no significant additional effects of asthma regarding sick leave (Fig 2 and Table 3). It is also possible to obtain data from the Social Insurance Agency, this was not available in this study. Moreover, sick leave for subjects with CRS is significantly increased across many, although not all, occupational groups, indicating a tangible effect of this disease on working life.

## Conclusion

CRS is a burdensome disease, and we show that afflicted subjects have a higher prevalence of sick leave across many occupational groups, as compared to subjects without CRS. Women in particular appear to be more affected. This underlines the importance of identification and adequate treatment of patients with CRS.
